# Epidemiology of schistosomiasis in the town of Manjo, littoral - Region,Cameroon

**DOI:** 10.1016/j.parepi.2023.e00319

**Published:** 2023-07-29

**Authors:** Yamssi Cedric, Gamago Nkadeu Guy-Armand, Noumedem Anangmo Christelle Nadia, Tako Djimefo Alex Kevin, Vincent Khan Payne

**Affiliations:** a Department of Biomedical Sciences, Faculty of Health Sciences, University of Bamenda, P.O. Box 39, Bambili, Cameroon; b Department of Animal Biology, Faculty of Science, University of Dschang, P.O. Box 067, Dschang, Cameroon; c Department of Microbiology, Hematology and Immunology, Faculty of Medicine and Pharmaceutical Sciences, University of Dschang, P.O. Box 96, Dschang, Cameroon; d Department of Animal Organisms, Faculty of Science, University of Douala, P.O. Box 24157, Douala, Cameroon

**Keywords:** Epidemiology, Risk factors, Parasite load, Manjo, Cameroon, *S. haematobium*, *S. mansoni*

## Abstract

**Background:**

Schistosomiasis is endemic in Cameroon and continues to cause serious public health problems, especially among populations in rural areas. This study aimed at determining the prevalence and risk factors of urinary and intestinal schistosomiasis in Manjo.

**Method:**

A cross-sectional study was conducted in the city of Manjo in 2020. Stool and urine samples were collected from 400 participants. These stool and urine samples were examined by the Kato Katz, and centrifugation methods respectively.

**Results:**

The results obtained showed an overall prevalence of 6.25%, with 5% and 1.25% for *S. mansoni* and *S. haematobium* respectively. A significant difference (*p* < 0.05) was revealed among occupations, age groups, neighborhood, water usage, educational level, knowledge of the disease meanwhile no significant difference was observed between gender and occupation according to prevalence. The most infected ages were] 50-; + [and]20–35] with 13.36% and 11.86% respectively. *S. haematobium* revealed a low infection intensity while *S. mansoni* showed moderate infection intensity. The mean parasite load for *S. haematobium* was 6 ± 3.225 Eggs/10 ml in females and 7 ± 4.243 Eggs/10 ml for males; while the mean parasitic load in *S. mansoni* was 180 ± 142.441 Epg in females and 146.67 ± 82.286 Epg in males.

**Conclusion:**

Manjo can be classified as a low endemic area with a prevalence rate of 6.25% and species observed were *S. haematobium* and *S. mansoni*. Also, risk factors where observed including the use of water from the river for domestic purposes. Therefore, the intensification of health education campaigns among the population would delay the development of this disease in the locality.

## 1 Background

Schistosomiasis, also known as bilharzia, is the second most common global parasitic disease in the world (Tchouanguem et al., 2016). It is an acute and chronic parasitic infection caused by worms (trematodes) of the genus *Schistosoma*. Sixteen species of schistosomes are distinguished, of which six are pathogenic to humans: *S. haematobium, S. intercalatum, S. guineensis, S. mansoni, S. japonicum,* and *S. mekongi* (Soko et al., 2017). Worldwide, approximately 600 million people in 78 countries in Africa, South America, the Middle East, and South Asia are at risk of contracting the disease (Mondiale de la Santé and World Health, 2016). Today, it is estimated to cause 251.4 million cases of disease and about 200.000 deaths (Mondiale de la Santé and World Health, 2016).

Africa alone bears the greatest burden, with over 90% of the world's infected people. Today, with the COVID-19 pandemic, efforts to mitigate the consequences have reduced the number of responders, thus increasing prevalence.

In Cameroon, the various national surveys conducted by the National Schistosomiasis Control Programme (PNLSHI) show that >5 million people, or 33% of the population, are at risk of infection, and currently 2 million people, or 13%, are parasitized (Dankoni and Tchuenté, 2014). Today, despite the efforts of stakeholders involved in the eradication of this disease, it remains a serious public health problem (Dakoni et al., 2015). The control strategy based on the control of intermediate host molluscs using molluscicide products and chemotherapy remains costly (Tchuenté, 2006). The real problem is that the town of Manjo does not have access to safe drinking water for most of its population. It is therefore forced to be in contact with contaminated water points, notably the Dibombé River (the river that runs through the town of Manjo) and the spring that the town relies on for its supplies, which is conducive to the spread of these diseases. As the diagnosis of these diseases is not done in a regular and systematic way, and especially as no similar study has been carried out in this zone, we find it necessary to know the state of health of the population of Manjo with regard to these schistosomiases in order to elaborate adapted control strategies.

## 2 Material and methods

### 2.1 Study site and study design

**Fig. 1:**
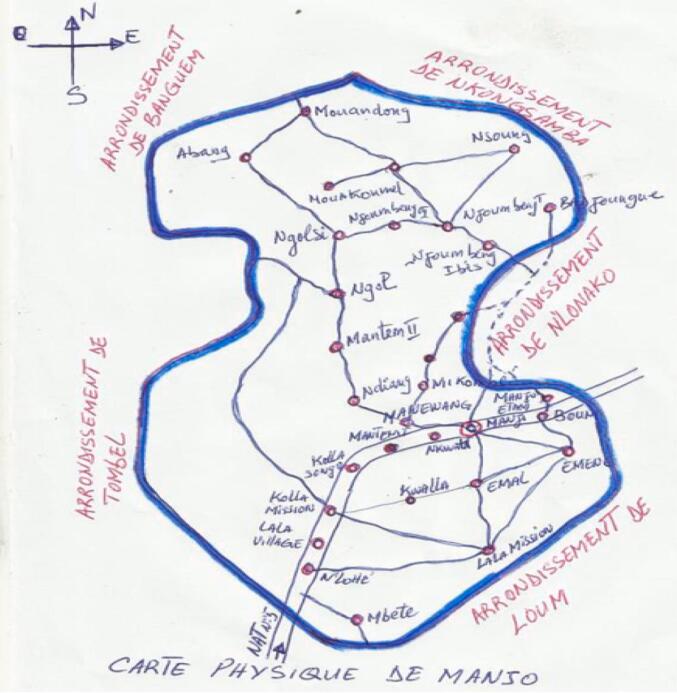
Map of Manjo.

A cross-sectional study was carried out during a period of 3 months from May to July where 5 randomly selected neighborhoods (Quarter) were selected in the city of Manjo located in the northern part of the Littoral Region, Cameroon (Fig. 1). Manjo covers an area of 305 km^2^ between 9° 41 and 9° 50 east longitudes and between 4° 42 and 4° 53 north latitude. Two seasons characterize the climate: a six-month rainy season (March–November) and a six-month dry season (November–March) (Kamga et al., 2022).

### 2.2 Study population

Four Hundred participants were recruited for this study and consisted mainly of pupils, students, housewives and traders from different part of the country. The town harbors two main health facility; a Confection catholic Hospital and a District Hospital. The main water sources used by the population comes from the spring and the Dibombé River. Regarding the campaigned distribution of praziquantel in the region, the latest dated 16 months before the beginning of the study.

### 2.3 Inclusion and exclusion criteria

Any person who had not received Praziquentel for >6 months and who signed the Informed Consent form or whose parent signed were included. Excluded from the study were person not residing in Manjo.

### 2.4 Sample size determination

The sample size was determined according to the Lorenz formula (StatCalc of EPI Info software). Using the prevalence of *P* = 20.1% from the work of Payne et al., 2019 in Njombé with an 80% power to detect significant associations or differences and a 5% accepted margin of error, the minimal sample size estimate was 400 participants.

### 2.5 Data collection procedure

The microscopic analyses were carried out by a team under the supervision of the Manjo District Hospital laboratory. After selecting a person who met the inclusion criteria, a questionnaire was first submitted. The first part consisted of questions on socio-demographic data (age, gender, occupation, level of education, neighborhood) while the second was on risk factors (water sports activities, knowledge of the disease). Then, the consent form was given to each person to sign. Finally, two sterile containers were given to them. One was for urine, and the other was for stool sample. The sampling techniques were explained to them.

### 2.6 Sample collection and parasitological examination

Data collection in Manjo city was done in five (5) randomly selected quarters (Quarter I, Quarter II, Quarter III, Quarter V and Quarter VI) out of the nine (9) quarters in the city, including schools, homes, and the Manjo District Hospital. Collection times ranged from 7:30 a.m. to 10:30 am. Participants who met the inclusion criteria were given two containers each for collection: one for stools and one for urine. The stool samples were kept cool during the duration of collection. The urine was preserved with 2–3 ml of 10% diluted formalin. The samples were then transported to the Manjo District Hospital laboratory for examination. The stools were examined by the Kato-Katz method (Katz et al., 1972). One stool was read per subject. As for the examination of urine by the centrifugation method, and the entire pellet was observed under a 40×. The studied parameters were: prevalence and intensity of infection.

$$\text{Prevalence}=\frac{\mathrm{Number of infected people}}{\mathrm{Total Number examined}}*100$$

Intensity = ∑ of eggs of parasite counted by slide *20 (50 mg perforated plate) (OMS, 1994).

### 2.7 Ethical approval and consent to participate

Before starting the study properly, the research proposal was submitted to the District Hospital Review Board (DHB) in Manjo with Registration N^O^ 21/APP/RDPH/DHB for evaluation and request for ethical clearance which was approved.

### 2.8 Statistical analysis

The data collected was entered into Microsoft Office Excel version 2010 software and then transferred to SPSS version 20.0 (Statistical Package for Social Sciences) software for statistical tests. The Chi-square test (*X*^2^) was used to compare the prevalence. ANOVA test for the intensity of infection and the Risk Test allowed us to calculate the Odds ratio to determine the risk factors. The tests were statistically significant at p ˂ 0.05.

## 3 Results

**Fig. 2:**
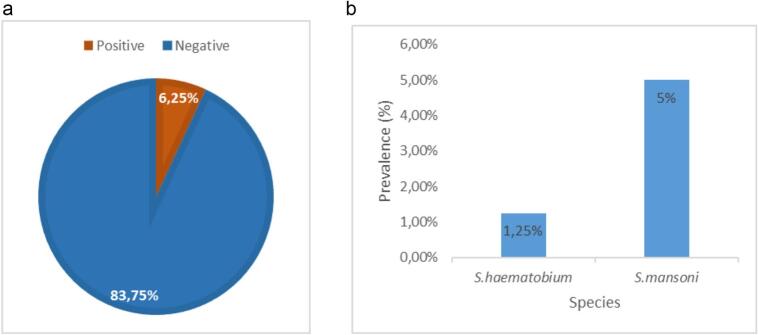
a. Overall prevalence. b. Prevalence according to species.

Fig. 2a and b show the overall prevalence of infection and the prevalence of different species observed; it can be seen from these figures that the overall prevalence was 6.25%. It was 5% and 1.25% for *S. mansoni* and *S. haematobium* respectively.

Table 1 shows the prevalence of infection according sex and age. According to this Table, a slight variation was observed between Male (8.44%) and Female (4.7%) with no statistical significant difference while a significant difference (*p* = 0.129) was observed among different age groups with the most infected groups being 50 years and above and]20–35[having a prevalence rate of 13.33% and 11.86% respectively (*p* = 0.02).

**Table 1:** Prevalence of schistosomiasis according to sex and age group.

Parameter	Total number examined	Number of positive cases (Prevalence (%)		Total Prevalence (%)	*p*-Value
		*S.haematobium*	*S. mansoni*		
Sex					0.129
Female	234	3 (1.28)	8 (3.42)	4.7%	
Male	166	2 (1.21)	12 (7.23)	8.44%	
Age (Years)					0.02
[0–5]	50	0 (0)	2 (4)	4	
[5–10]	62	0 (0)	1 (1.61)	1.61	
[10–20]	88	0 (0)	4 (4.55)	4.55	
[20–35]	118	4 (3.39)	10 (8.85)	11.86	
[35–50]	67	1 (1.49)	1 (1.49)	2.99	
[50-and +]	15	0 (0)	2 (13.33)	13.33	
Total	400	5 (1.25)	20 (5)	6.25	

According to Table 2, prevalence does not vary according to occupation (*p* = 0.153). Also, workers in the informal sector (7.69%) seem more infected than pupils/students (5.49%) and workers in the formal system (3.030%).

**Table 2:** Prevalence with respect to job occupation.

Occupations	Number examined	*S. haematobium*		*S. mansoni*	
		Number of positive cases	Prevalence (%)	Number of positive cases	Prevalence (%)
Pupils/Students	242	02	0.83%	11	4.54%
Formal sector worker	33	0	0%	02	3.030%
Informal sector worker	112	03	2.75%	08	7.14%
Total	400	05	1.25%	20	5%
P.value		0.225		0.448	

Formal sector worker: those who carry out economic activities in the informal sector (civil servant, company employee) Informal sector worker: those who carry out economic activities in a structured sector (hairdresser, dressmaker).

Table 3 shows the prevalence of infection according to water-related activities and quarters. According to this Table, a significant difference (*p* = 0.01) was observed based on water-related activities. The highest prevalence was recorded among those who bathed in the river (46.15%) and among those who did laundry (43.33%). Also, prevalence was higher among those who frequently visited the Dibombè River (28.88%) compared to those who frequently visited the source (Tap water) (8.22%). Also, prevalence varied according to quarter (p = 0.01). The prevalence was higher in Quarter II (16.2%) compared to Quarters V and VI, both having a prevalence of 7.5%.

**Table 3:** Prevalence of schistosomiasis by neighborhood and water-related activity.

Parameters	Number examined	Number of positive cases	Prevalence (%)	*P* value
Activities in water bodies				0.01
No activity	197	00	0%	
Laundry	30	13	43 0.33%	
Bathing	13	6	46 0.15%	
Carry water	140	4	2.77%	
Dipping of legs in water	14	2	12.5%	
Quarters				0.01
Quarter II	80	13	16.25%	
Quarter V	80	06	7.5%	
Quarter I	80	00	00	
Quarter III	80	00	00	
Quarter VI	80	06	7.5%	

Table 4 shows the prevalence of schistosomiasis with respect to the level of education and knowledge about the disease. The prevalence varies with a significant difference (*p* = 0.01) according to the level of education. Illiterates were the most infected (23.52%), while persons who had attained university education were the least infected (2.94%). Likewise, prevalence varied according to knowledge of the disease (*p* = 0.01).

**Table 4:** Prevalence of schistosomiasis according to the level of education and knowledge of the disease.

Parameters	Number examined	Number of positive cases	Prevalence (%)	P value
Level of education				0.01
Illitrates	17	04	23.52	
Primary	168	16	2.95	
Secondary	181	10	5.52	
University graduate	34	01	2.94	
Knowledge of the disease				0.01
No	396	22	5.5	
Yes	04	03	75	

Knowledge of the disease: have heard of schistosomiasis and know the mode of transmission.

Table 5 shows the average parasite load of *S. haematobium*. According to this Table, the parasite load of *S. haematobium* did not vary with sex (*p* = 0.60), but a slight increase was observed in female participants (7 ± 4.243eggs/10 mL) compared to male participants (6 ± 3.225 eggs/10 mL). Parasite load for *S. mansoni* also did not vary with gender (*p* = 0.97). The mean parasite load of *S. mansoni* appears to be higher in females (180 ± 142.441 Epg) than in to males (146.67 ± 82.286 Epg) but this difference was not statistically significant. Similarly, the mean parasite load of *S. haematobium* appears to be higher in pupils/students (7.5 ± 3.536 eggs/10 mL) compared to workers in the informal sector (5.67 ± 3.786 eggs/10 mL). This also shows that the mean parasite load of *S. mansoni* appears to be higher in workers in the informal sector (148.57 ± 74.706 Epg) compared to pupils/students (145. ± 133.519 Epg) and those in the informal sector (100 Epg). Parasitic infections did not varied according to occupation (*p* > 0.05). It follows that the highest average parasite load with *S. haematobium* was recorded in the]20–35] age group with (7.25 ± 3.202 Eggs/10 mL) while the lowest was the]35–50] group with (3 Eggs/10 mL). The parasitic infections with *S.mansoni* and *S.haematobium* did not vary statistically according to the age group (p > 0.05).

**Table 5:** Average parasite load of *S. mansoni* and *S. haematobium* according to Sex.

	Species	
	*S. mansoni*	*S. haematobium*
Sex		
Female	180 ± 142.441 Epg	7 ± 4.243 Eggs /10 ml
Male	146.67 ± 82.286 Epg	6 ± 3.225 Eggs /10 ml
Occupation		
Informal sector worker	148.57 ± 74.706 Epg	5.67 ± 3.786 Eggs/10 ml
Students	145. ± 133.519 Epg	7.5 ± 3.536 Eggs/10 ml
Formal sector worker	100 Epg	0 Eggs/10 ml
Ag group		
[0–5]	150.00 ± 70.711 Epg	0 Eggs/10 ml
[5–10]	100	0 Eggs/10 ml
[10–20]	200 ± 200Epg	0 Eggs/10 ml
[20–35]	141.82 ± 86.466 Epg	7.25 ± 3.202 Eggs/10 ml
[35–50]	0	3 Eggs/10 ml
[50-and +]	200 Epg	0 Eggs/10 ml

### 3.1 Risk factors

Table 6 shows risk factors associated with sex, water-related activities, knowledge of the disease, age, and level of education. According to this Table, laundry (OR = 5.35 at 95% CI at [27.823–1.030]) and bathing (OR = 6.00 at 95% CI at [37.76–0.95]) were the main risk factors, unlike those who come to fetch water and those who dip their legs into water. Equally, the age group] 20–35] was more at risk (OR = 2.17 at 95% CI at [0.93–5.02]) and a significant difference was observed between age group and Schistosoma infection. Education level was not considered a risk factor (*p* > 0.05), whereas knowledge of the disease was a risk factor (OR = 51.00 at 95% CI [51.00–510.55]). There was a significant relationship between knowledge of the disease and infestation (p ˂0.05). Furthermore, females were not at a higher risk of contracting the disease (OR = 0.536 at 95% CI [1.211–0.231]).

**Table 6:** Risks factors.

Risk Factors	Number examined	Positive cases	Odds ratio	CI 95%	P-value
Sex					
Female	234	11	0.536	1.21–0.23	0.134
Male	166	14	–	–	–
Age group					
[0–5]	50	02	3692	28.78–0.47	0.212
[5–10]	62	01	9.385	111.39–0.79	0.076
[10–20]	88	04	3.231	19.44–0.53	0.200
[20–35]	118	14	1.143	5.60–0.23	0.042*
[35–50]	67	02	5.000	38.77–0.64	0.124
[50- et plus]	15	02	–	–	–
Level of education					
Illiterates	17	04	0.677	4.51–0.10	0.650
Primary	168	10	1.539	5.91–0.40	0.530
Secondary	181	10	1.655	6.35–0.41	0.463
University	15	0	–	–	–
Occupation					
Students /Pupils	242	13	2.048	4.72–0.88	0.093
Formal Sector	33	01	3.630	29.19–0.45	0.225
Informal sector	100	11	–	–	–
Knowledge of the disease					
Yes	04	03	–	–	–
No	396	22	51.00	510.54–5.09	0.001*
Quarters					
Quarter II	80	13	0.42	6.65–0.86	1.000
Quarter I	80	00	0.00	0.00	–
Quarter III	80	00	0.00	0.00	–
Quarter VI	80	06	1.00	3.24–0.30	0.094
Quarter V	80	06	–	–	–
Activities in water bodies					
Laundy	30	13	5.35	27.82–1.03	0.041*
Bathing	13	06	6.00	37.76–0.95	0.046*
Carry water	140	04	0.20	1.19–0.34	0.07
No activity	197	00	0.00	0.00	–
Dipping of legs in water	14	02	–	–	–
Frequency of visits to rivers/Springs					
Tap	350	10	1.66	5.91–0.47	0.2
Dibombe River	45	13	20.00	67.16–6.19	0.7
Springs	146	12	–	–	

## 4 Discussion

The overall prevalence of Schistosoma infection in this study was 6.25%, with 1.25% for *S. haematobium* and 5% for *S. mansoni*. These results show the low endemicity of urogenital and intestinal schistosomiasis, as the prevalence is <10% (Saotoing et al., 2014). The same observations were made by Saotoing et al. (2016) in Maga subdivision, where they obtained 2.2% (*S. mansoni*), and by Dankoni and Tchuenté (2014) in Kekem subdivision, West Cameroon, where they obtained 1.7% (*S. haematobium*). However, these observations differ from earlier studies by Saotoing et al. (2014) and Payne et al. (2019), who respectively obtained a prevalence of 38.5% in a study in the Mayo-Louti subdivision of Far North Cameroon and 20.1% in a study in the Djombe subdivision. The low prevalence obtained in our work can be explained not only by the fact that a mass Praziquantel treatment and sensitization campaign had been organized by the National Programme for the Control of Intestinal Schistosomiasis and Helminthiasis in Cameroon (PNLSHI) 16 months before the work was carried out but also by the technique of identifying eggs in urine, which is less sensitive. No significant difference was observed between Schistosome infections with respect to sex (*p* > 0.05). Males had a higher prevalence (8.44%) compared to females (4.70%). This could be justified by the fact that men are involved in water-related activities than women and therefore are more predisposed to infection with this parasite. These results corroborate those of Randrianasolo et al. (2015) in Madagascar where there was no significance with respect to gender for schistosome infection (*p* > 0.05). He reported that males accounted for 64.3% of the infection. Also, a study conducted by Dankoni and Tchuenté (2014) in the Kekem District revealed no statistically significant difference between sexes in schistosome infection when they had a prevalence of 2.5% in males and 1.0% in females. On the other hand, the results of Saotoing et al. (2016) and Senghor et al. (2014) contradict it. They found a significant difference in schistosome infection according to gender in their study with females having a higher prevalence.

Likewise, no statistically significant difference was observed between age groups. Higher prevalence was observed only among adults' participants in the age group]20–35] years old with 11.86%, and 13.35% in person 50 years above. However, in the 0–20 years old of group prevalence was low. This could be justified by the fact that unlike young participants, adults are participated in the deworming and free treatment campaigns. A similar study conducted by Dakoni et al. (2015) in Taibong subdivision in Far North Cameroon also a low prevalence in young participants 0–14 years. On the other hand, studies carried out by Senghor, (2010) in Niakhar (rural area) in Senegal by Alebie et al. (2014) in Ethiopia, presented young participants ([0–20 [years) as the most infected age group.

On the other hand, there is a significant difference (*p* < 0.05) in the prevalence of *Schistosoma* infection according to quarters. This is justified by the fact that the inhabitants of these areas preferentially use river water or those from the spring for domestic use to reduce water bills thereby increasing human contact with water. Also, the non-awareness of the disease and its route of transmission by the majority of participants predisposed them to the infection. A similar study carried out by Senghor, (2010) in rural areas of Senegal (Niakhar) showed a high prevalence in some neighborhoods but low in others. He justified the high prevalence by the absence of a drinking water distribution network. On the other hand, a study carried out in an urban area in Mélen (Yaoundé-Cameroon) showed mostly low infections in the neighborhoods. The authors justified this by the fact that urbanization effectively reduces transmission points and the creation of modern water points limits human-water contact (Njiokou et al., 2004). Our results show that illiterates had a high prevalence (23.52%) of *Schistosoma* infections with respect to the level of education. Indeed, the lower the level of education, the higher the prevalence. The differences observed between the levels of education were statically significant (*P* < 0.05). This could be linked to the fact that in many be hygiene was taught in nursery school and their understanding is done throughout our school career. As a result, parents with no or less education will not be able to teach their children good hygiene. A similar study carried out by the Nestlé Company in Ivory Coast also revealed this (Crompton, 2006). On the other hand, the study by Payne et al. (2019) in Djombe presented illiterate participants as the least infected.

No significant difference was observed with respect to occupation. However, a higher prevalence was recorded in housewives (40%) and among those who were involved in agro-pastoral activities (18%). This could be explained by the fact that the latter are regularly involved contact with water contact activities either when they are doing their work in the fields or when they go to do laundry in springs or rivers. A similar observation was made by Traoré (2020) in the Sanitary District of Bougouni, Bankass and Tominian presenting a high risk of contracting schistosomiasis for those who carried out an agro-pastoral activity compared to those with other professions. Payne et al. (2019) reported a contrary observation when they presented farmers as the least infected (1.38%).

Surprisingly, the prevalence was higher among those who said “Yes” to know the disease (74.45%) compared to those who did not know the disease (25.55%). This high prevalence could be justified by the very small sample size of those who said they knew the disease. The same observation was made by Randriamiharimanana (2017) who observed a higher prevalence in those who knew the disease (15.5%) compared to those who did not (11.1%).

Differences in mean parasitic loads were not statistically significant with respect to sex for *S. haematobium* infestation. The mean parasitic load on *S. haematobium* was low (OMS, 2016). However, we found a higher mean parasitic load which seemed higher in female subjects (7 ± 4.243 Eggs /10 mL) compared to male subjects (6 ± 3.225 Eggs /10 mL). Similar studies by Kimbi et al. (2013) in Cameroon in the South-West region and by Payne et al. (2019) presented a higher mean parasite load in female subjects compared to male subjects. Likewise, no significant difference with respect to gender for *S. mansoni* infection. But the parasitic load appeared to be greater in females (180 ± 142.441 Epg) compared to males (146.67 ± 82.286 Epg). This may be justified by the fact that females are more regularly in contact with water than males. Our results corroborate with the results of Anto et al. (2014) who had a higher mean parasite load in females compared to males. On the other hand, studies conducted by Ibikounlé et al. (2014) in Benin and by Kimbi et al. (2013) in Barombi in the South West region, Cameroon showed a higher parasite load in males. According to our study,water -related activities such as swimming (OR = 16.602 at 95% CI to [5, 18–54.24]), laundry (OR = 22.814 at 95% CI to [0.64–3.26]) were risk factors. A statistically significant association was observed between these two risk factors for schistosome infection (*p* < 0.05). This reflects a strong association between these risk factors and schistosome infection. A similar study by Randriamiharimanana, (2017) in Madagascar where not only present swimming in ponds was presented as a risk factor associated with schistosomiasis (OR = 2.20 at 95% CI to [1.59–9.68] but there was also a statistically significant association between swimming in ponds and infection with schistosomes (*p* < 0.05). Likewise, our study shows that the frequent visit of river Dibombè river (OR = 11.612 at 95% CI to [4.89–27.57]) unlike springs (OR = 1.45 at 95% CI to [4.89–27.57]) presents a risk factor. Statistically, a significant association was noted between the frequency of visiting the Dibombe river and schistosome infection (p < 0.05). Similar observation was made by Randriamiharimanana, (2017) in Madagascar frequent visiting of ponds was a risk factor. The risk of being infected was greater in the 0–50 age group compared to those aged 50 and above. Similarly, Odiere et al. (2012) in their work in the town of Kabana found adult subjects to be at greater risk of contracting the disease. This is justified by the fact that at this age people are more active and therefore frequent schistosome infestation sites.

## 5 Conclusion

Manjo can be classified as a low endemic area for schistosomiasis with a low overall prevalence of 6.25% and the species *S. haematobium* and *S. mansoni* were observed. Risk factors including the use of water from the river were documented. Intensification of health education campaigns in the population to bring awareness is very strongly advocated.

## Availability of data and materials

Data and material are available to other researchers upon request.

## Funding

No external fund.

## Author's contributions

GNGA, VKP, YC, NACN and TDAK contributed to the design of the study, data collection, led the analysis and drafting of the manuscript. All authors read and approved the final manuscript.

## Consent for publication

Not applicable.

## Declaration of Competing Interest

The authors declare that they have no competing interests.
